# Selenium Nanoparticle Activity against *S. mutans* Biofilms as a Potential Treatment Alternative for Periodontitis

**DOI:** 10.3390/pharmaceutics16040450

**Published:** 2024-03-25

**Authors:** Naasika Hamman, Poornima Ramburrun, Admire Dube

**Affiliations:** 1 Infectious Disease Nanomedicine Research Group, School of Pharmacy, University of the Western Cape, Bellville 7535, South Africa; 3635984@myuwc.ac.za; 2 Wits Advanced Drug Delivery Platform Research Unit, Department of Pharmacy and Pharmacology, School of Therapeutic Sciences, Faculty of Health Sciences, University of the Witwatersrand, Johannesburg 2193, South Africa; poornima.ramburrun@wits.ac.za

**Keywords:** selenium nanoparticles, periodontitis, biofilms, dentistry, *S. mutans*, biofilm inhibition

## Abstract

The disruption of periodontal biofilms and prevailing antimicrobial resistance issues continue to pose a great challenge to the treatment of periodontitis. Here, we report on selenium nanoparticles (SeNPs) as a treatment alternative for periodontitis by determining their antibiofilm activity against *S. mutans* biofilms and the potential role of particle size in disrupting biofilms. SeNPs were synthesised via a reduction reaction. Various physicochemical characterisations were conducted on the NPs, including size and shape. The microbroth dilution method was used to conduct the biofilm and antibiofilm assay against *S. mutans*, which was analysed by absorbance. SeNPs displayed hydrodynamic sizes as low as 46 ± 4 nm at a volume ratio of 1:5 (sodium selenite/ascorbic acid) with good monodispersity and stability. Hydrodynamic sizes of SeNPs after resuspension in tryptic soy broth supplemented with 2.5% sucrose (TSB + 2.5% suc.) and incubated at 37 °C for 24 h, ranged from 112 to 263 nm, while the zeta potential values increased to greater than −11 mV. The biofilm assay indicated that *S. mutans* are weakly adherent, bordering on moderately adherent biofilm producers. The minimum biofilm inhibitory concentration (MBIC) was identified at 500 µg/mL. At a 1000 µg/mL concentration, SeNPs were able to inhibit *S. mutan* biofilms up to 99.87 ± 2.41% at a volume ratio of 1:1. No correlation was found between antibiofilm activity and particle size; however, antibiofilm activity was proven to be concentration-dependant. SeNPs demonstrate antibiofilm activity and may be useful for further development in treating periodontitis.

## 1. Introduction

Periodontitis is an accelerated and severe stage of gingivitis in which the gums become inflamed and detach from the teeth. This progressive disease can result in potential bone loss, tooth decay, and fallout [1]. The Global Burden of Disease Study, assessing disease burden from 1990 to 2016, revealed that periodontitis is the eleventh most prevalent disease around the globe and affects approximately 20–50% of the population [2], while severe periodontal diseases affect around 19% of adults globally [1]. Considered one of the biggest threats to dental health, periodontitis is a major public health concern that may hinder or interfere with the mastication process and alter one’s appearance and confidence, consequently affecting quality of life [3]. Periodontal diseases may similarly expose people to vast socio-economic bearings and healthcare costs. In 2018, a study intended to estimate the economic burden of periodontitis in the US and Europe revealed that indirect costs as a result of periodontal disease totalled USD 150.57 billion and EUR 156.12 billion in Europe. The overall estimates also revealed that periodontal disease caused a total loss of USD 154.06 billion in the US and EUR 158.64 billion in Europe in the year 2018, of which indirect costs made a significant impact [4].

The oral cavity is an intricate ecosystem which hosts over 150 diverse bacterial species in an individual, as well as other types of microorganisms, including arches, fungi, protozoa, and viruses [5]. The onset of periodontitis is attributed to the formation of pathogenic biofilm. Biofilms are a complex network of bacteria that develop over time on dental surfaces [6], which can stimulate an inflammatory host response, resulting in the degradation of supporting periodontal tissues and subsequent tooth loss [7]. *Streptococcus mutans* is a Gram-positive bacterium that is an influential etiologic agent in dental caries [8]. *S. mutans* naturally thrive within the human oral cavity but more so in dental plaque, which is a biofilm of different species of microorganisms that develop on the hard surfaces of the tooth. This microbial species is not solely responsible for producing dental caries; it also plays a role in altering the local environment by developing a milieu that is rich in extracellular polysaccharides and low in pH [8,9]. These modifications, in turn, create an ideal environment for other acidogenic and aciduric species to flourish. Studies have been employed to detect *S. mutans* in oral sites, not only for its role in developing caries but also due to its association with extraoral pathologies. *S. mutans* and a subset of its strains have previously been associated with sub-acute bacterial endocarditis as well as some other extraoral infections such as cerebral microbleeds, immunoglobulin A nephropathy and atherosclerosis [10].

To date, no standardised treatment exists for periodontitis. However, the conventional course of treatment generally involves the implementation of non-surgical procedures such as debridement, scaling and root planning (SRP). These non-surgical procedures eradicate plaque biofilm and calculus, followed by smoothing the tooth or root surface and may be performed both supra- and sub-gingivally [11]. While SRP is reported to be successful at reducing the microbial load within the oral cavity, it has not proven efficacious at eliminating the pathogenic species from a subject infected with periodontitis and may result in the recolonisation of these species and possibly the progression of periodontitis [12]. Therefore, these procedures typically require the use of adjunctive antimicrobial and anti-inflammatory agents. However, these chemotherapeutic agents are not short of limitations. Studies have previously revealed high rates of resistance of selected sub-gingival periodontal pathogens to a broad range of antimicrobial agents typically used in clinical practice [13]. Hence, supplementary microbial and antibiotic susceptibility testing prior to initiating antimicrobial therapy may be required. Also included in the potential treatment options is the development of advanced local drug delivery devices such as films, fibres, gels, and strips. However, the difficulty in gaining access and determining the depth perception of periodontal pockets, costly manufacturing, the use of non-bioabsorbable and non-biodegradable materials, and troublesome and time-consuming insertion and removal of certain devices make these advanced drug delivery options a challenging therapeutic alternative [14].

Nanotechnology promises to provide an opportunity to resolve certain shortcomings experienced by current treatment modalities, especially those involving the treatment of periodontitis. The field of nanotechnology offers treatment alternatives for the restoration of damaged, infected, absent, and fractured teeth [15,16]. Several of the latest progressions in this field include the integration of nanocomposites, nanoimpressions, and nanoceramics within clinical dentistry [15]. Metallic nanoparticles (NPs) are small particles that are 1–100 nm in size and are composed of merely a few hundred atoms. Metallic NPs, along with their innate antibacterial activity, present several possibilities for eliminating pathogenic microorganisms in the oral cavity in comparison to conventional treatment [17]. Along with antibiofilm and antibacterial activity, metallic NPs may also play a regenerative role within the oral cavity [18]. The biggest advantage of metallic NPs as antimicrobial agents is their ability to simultaneously act through multiple mechanisms. The many possible mechanisms include stimulating reactive oxygen species (ROS) production, which may hinder DNA replication and amino acid synthesis, resulting in the destruction of the bacterial cell membrane [19]. As a result, microbes are unable to develop resistance to these mechanisms of action, contrary to conventional antibiotics [20]. Selenium nanoparticles (SeNPs) have gained a considerable amount of attention as they possess favourable properties such as biocompatibility, bioavailability, and low toxicity and have been proven to bear excellent antimicrobial activity [21,22]. With the imminent rise in antimicrobial resistance and the need for alternate antibacterial agents, metallic NPs have proven to be effective at combating microbial infections as their bactericidal activity is different from those of conventional antimicrobial agents [23,24]. Although studies have already proven that SeNPs exhibit exceptional antimicrobial activity, limited research explores their antibiofilm activity against *S. mutans* and whether the size of the NPs could influence biofilm penetration and, consequently, biofilm disruption. This work, therefore, aimed to synthesise and characterise SeNPs and determine the antibiofilm activity and MBIC of SeNPs against *S. mutans* while also distinguishing the effect of particle size on antibiofilm activity.

## 2. Materials and Methods

### 2.1. Materials

Sodium selenite (Na_2_SeO_3_), ascorbic acid (C_6_H_8_O_6_), sodium dodecyl sulphate (SDS) (NaSO_4_C_12_H_25_), sodium chloride (NaCl), acetic acid (CH_3_COOH), sucrose, Petri dishes and 96-well plates were acquired from Sigma-Aldrich (St Louis, MO, USA). Tryptic soy broth (TSB) and tryptic soy agar (TSA) were purchased from Merck (Darmstadt, Germany). *S. mutans* (ATCC 25175) were obtained from Thermo Fisher Scientific (Johannesburg, South Africa). Crystal violet (CV) solution was purchased from DLD Scientific (Durban, South Africa). Ethanol, methanol and acetic acid were purchased from Laborem (Cape Town, South Africa). Ultra–purified distilled water (18.2 MΩ cm, Millipore, Merck, Darmstadt, Germany) was used in all experiments.

### 2.2. SeNP Synthesis

SeNPs were produced via a wet chemical reduction reaction, which was adapted from [25,26] with necessary modifications. A quantity of 0.179 g of sodium selenite was transferred to a conical flask along with 10 mL of 10 mM SDS stock solution and stirred at 300 rpm, at 25 °C for 30 min. While the reaction proceeded, various volumes of 50 mM ascorbic acid stock solution were added dropwise to produce multiple samples with final volume ratios of 1:1, 1:3 and 1:5 of sodium selenite to ascorbic acid. The reaction proceeded for 30 min, and colour changes were observed from clear to orange/red. Thereafter, each NP sample was centrifuged for 40 min at 12,580× *g*. The precipitate was collected and redispersed in dH_2_O. This experiment was repeated and performed in triplicate for each volume ratio (*n* = 3). The SeNP samples were stored at 4 °C for future use. Bulk stock solutions of SeNPs with known concentrations were prepared by drying, weighing, and resuspending samples from each volume ratio in sterile water and, thereafter, sonicating. Bulk samples were also stored at 4 °C for future use.

### 2.3. Characterisation of SeNPs

#### 2.3.1. Hydrodynamic Size, Polydispersity Index and Zeta Potential Analysis

The hydrodynamic size, polydispersity index (PDI) and ZP of the SeNPs were characterised using Zetasizer ZS-90, which employs the dynamic light scattering (DLS) technique (Malvern, UK, software version 8.00.4813). NP samples were prepared as described in Section 2.2. *SeNP Synthesis*, after which SeNP samples were redispersed in dH_2_O and temporarily refrigerated (˂24 h) before being subjected to size, PDI and ZP analysis at an unknown concentration. Each sample was pipetted into a disposable polystyrene cuvette for size and PDI and a DTS70 cell for ZP analysis at 25 °C.

#### 2.3.2. High-Resolution Transmission Electron Microscopy (HR-TEM) Analysis and Size Distribution

HR-TEM analysis was performed on SeNP samples at volume ratios of 1:1, 1:3 and 1:5 in dH_2_O, which were analysed using a FEI Tecnai F20 TEM (Thermo Fisher (FEI), Eindhoven, The Netherlands) at 200 kV. The core diameter of the SeNPs was verified via Image J software version 1.53k from the TEM images acquired, and the particle size distribution graphs were constructed on OriginPro 2023b software.

#### 2.3.3. Stability of SeNPs

The stability of SeNPs was evaluated in dH_2_O and in TSB + 2.5% suc. with a final concentration of 1 mg/mL. The hydrodynamic size, PDI and ZP were measured at 0-, 1-, 7-, 14- and 30-daytime periods using DLS (Malvern Zetasizer, software version 8.00.4813). Samples of 1 mg/mL concentration of SeNPs in TSB + 2.5% suc. were incubated for 24 h at 37 °C and characterised via DLS.

#### 2.3.4. *S. mutans* Culture Preparation

Approximately 3–4 single colonies were selected from the overnight culture of isolated *S. mutans* and used to inoculate a 5 mL tube containing sterile TSB + 2.5% of suc., which was then incubated overnight at 37 °C. The overnight culture was adjusted to obtain an optical density (OD) equivalent to 0.5 McFarland using a spectrophotometer at a wavelength of 540 nm. The bacterial suspension was then further diluted to 1:100 with TSB + 2.5% suc. The wells on the perimeter of a 96-well plate were filled with 200 µL of sterile water to avoid possible contamination. The remaining wells contained 200 µL aliquots of bacterial suspension and uninoculated TSB + 2.5% suc. (sterility controls). The plates were then sealed and incubated overnight at 37 °C for biofilm formation. The assay was repeated in triplicate for an incubation period of 24 and 48 h.

#### 2.3.5. Crystal Violet Biofilm Assay

After incubation, wells were decanted, and each plate was washed thrice with a sterile 0.9% saline solution. Biofilms that remained adherent after the wash were fixed with methanol at room temperature for 20 min, followed by the decanting of the methanol and air-drying of the plates. Test wells were then stained with 200 μL of a 0.1% CV solution for 15 min at room temperature. The CV solution was then discarded, and plates were washed thrice in sterile saline solution and allowed to dry. Adhered biofilm was resolubilised with 33% acetic acid. A sample from each test well was then transferred to the wells of a clean, optically clear 96-well plate. The absorbance was measured via UV spectroscopy (POLARstar Omega plate reader (software version 3.31), BMG LABTECH, Ortenberg, Germany) at a wavelength of 540 nm. The average and SD of the absorbance measurements were reported. The extent of biofilm adherence was categorised as shown in Table 1. The OD of the experimental wells containing inoculated media (OD) were compared to the OD of the sterility control (OD (control)) containing uninoculated media.

#### 2.3.6. Antibiofilm Assay

An overnight culture of *S. mutants* was adjusted to 0.5 McFarland and diluted to 1:100 in TSB + 2.5% suc. as similarly produced in the biofilm assay. A twofold serial dilution of UV-sterilised (via a UV chamber, Ultra-Violet Products, Inc., Los Angeles, CA, USA) SeNP suspension with a concentration of 2000 µg/mL and aliquots of TSB + 2.5% suc. was executed. The final concentration in test wells ranged from 7.81 to 1000 µg/mL. A 100 µL volume of the 100-fold bacterial suspension was pipetted into the test wells. Two separate columns were assigned as the growth and sterility control, which contained inoculated and uninoculated media, respectively. The wells on the perimeter of the plate were filled with sterile water. The plates were then covered, sealed and incubated overnight at 37 °C followed by further processing as per the CV assay described earlier. This experiment was repeated for each SeNP formulation ratio (1:1, 1:3 and 1:5) and then replicated thrice for each volume ratio to obtain a mean and SD (*n* = 3).

The percentage of inhibition of biofilm formation was calculated as shown in Equation (1) [28]. Biofilm inhibition was evaluated between 0 and 100%. Percentage inhibition below 0 was categorised as biofilm growth enhancement; values between 0 and 50% indicated weak antibiofilm activity, and above 50% depicted good biofilm inhibition [29].

Percentage of inhibition = [(OD_(Untreated)_ − OD_(Treated)_/OD_(Untreated)_)] × 100 (1)

#### 2.3.7. Statistical Methods

Measurements were reported as the mean ± standard deviation SD. Statistical analysis was measured via GraphPad Prism version 9.50 (730) using Tukey’s multiple comparison test (one- or two-way ANOVA), whereby probability values <0.05 were considered significant.

## 3. Results and Discussion

### 3.1. Hydrodynamic Size, PDI and ZP

**Figure 1:**
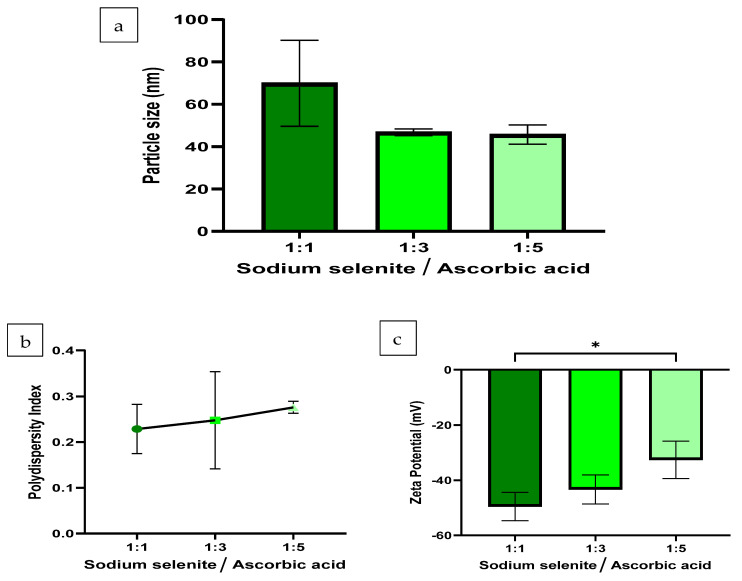
The (**a**) particle size, (**b**) PDI and (**c**) ZP results of the SeNP formulations at volume ratios of 1:1, 1:3 and 1:5 of sodium selenite to ascorbic acid (*n* = 3). The synthesis reaction proceeded at 25 °C, for 30 min, at 300 rpm and incorporated 10 mM of SDS as a stabiliser. Statistical significance is represented as follows—*: *p*-value ≤ 0.05. Error bars represent SD.

The average hydrodynamic sizes of SeNPs at volume ratios of 1:1, 1:3 and 1:5 were 70 ± 17 nm, 47 ± 1 nm, and 46 ± 4 nm, respectively (Figure 1a). Similarly, the PDI values at volume ratios of 1:1, 1:3 and 1:5 were 0.23 ± 0.04, 0.25 ± 0.09, and 0.28 ± 0.01, respectively (Figure 1b), indicating relatively monodisperse samples. The results indicate that the size of the SeNP is minimally affected by the proportion of the reducing agent in the formulation. Earlier studies on SeNP synthesis that tested similar volume ratios reported that the increase in the reducing agent during synthesis has a significant impact on the hydrodynamic size of the formulation [30,31]. In theory, ascorbic acid with a reducing capacity of 1–2, should fully reduce a volume ratio of 1:2 of sodium selenite to ascorbic acid; however, an excess of reducing agent causes greater reduction to occur, which subsequently retards oxidation and, therefore, results in smaller sized particles [31]. This concept is displayed by NP formulations with volume ratios of 1:3 and 1:5. SeNPs at volume ratios of 1:1, 1:3 and 1:5, and generated ZP values of −49.5 ± 4.2 mV, −43.3 ± 4.3 mV, −32.6 ± 5.56 mV (Figure 1c), respectively. As these formulations were observed to be relatively stable, no trend was detected between the reaction components and ZP, as no statistically significant data were produced in this particular experiment. It can, therefore, be deduced that the ZP and stability of the SeNP sample do not depend on the proportion of the ascorbic acid (the reducing agent) present during synthesis.

### 3.2. HR-TEM Analysis and Size Distribution of SeNPs

**Figure 2:**
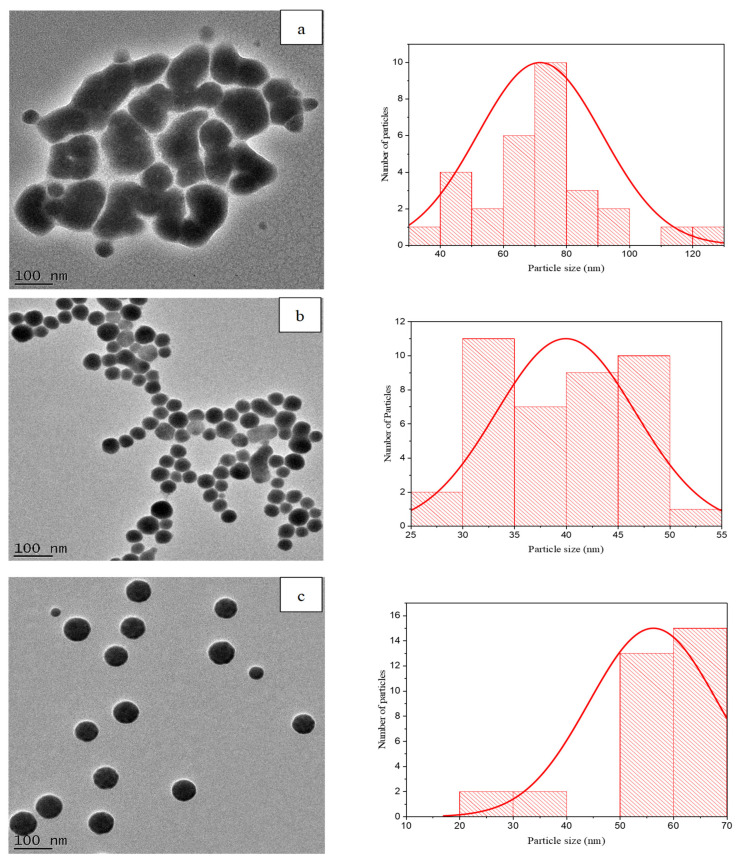
HR-TEM images displaying the morphology, core diameter and corresponding size distribution graph generated via Image J software for SeNPs suspended in dH_2_O at volume ratios (**a**) 1:1, (**b**) 1:3 and (**c**) 1:5 at 100 nm.

The TEM images presented in Figure 2a confirm that the 1:1 SeNPs had irregular-shaped particles, while the 1:3 (Figure 2b) and 1:5 (Figure 2c) SeNP samples displayed more uniformly shaped spherical particles with good monodispersity. It has been reported in the literature that increasing the reducing agent during the synthesis of certain metal-based NPs reduces the number of dispersed metallic nanoparticles, which subsequently reduces the agglomeration of nanoparticles, which seems to be the case in this study [32,33]. The size distribution graphs reveal that the average core diameter for the 1:1, 1:3 and 1:5 samples were 72 ± 20 nm, 40 ± 7 nm and 56 ± 12 nm, respectively, and these are comparable to the hydrodynamic diameters. No statistical differences were observed between the core diameter and the hydrodynamic diameters for each formulation ratio (*p* > 0.05).

### 3.3. Stability of SeNPs in dH_2_O

**Figure 3:**
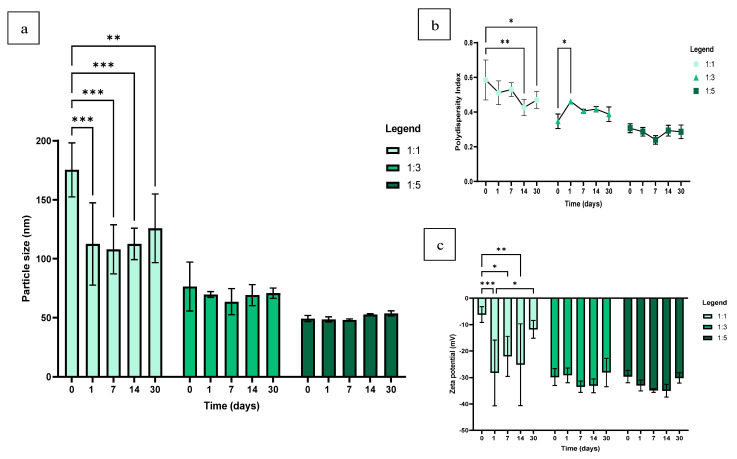
Stability of SeNPs in dH_2_O at different periods of 0-, 1-, 7-, 14- and 30 days and the effect the time had on the (**a**) particle size, (**b**) PDI and (**c**) ZP of SeNPs at different volume ratios of sodium selenite to ascorbic acid (*n* = 3). The synthesis reaction proceeded at 300 rpm, for 30 min, at 25 °C and incorporated 10 mM of SDS as a stabiliser. Statistical significance is represented as follows—*: *p*-value ≤ 0.05, **: *p*-value < 0.01 and ***: *p*-value < 0.001 Error bars represent SD.

The 1:3 and 1:5 volume ratios generated particle sizes well below 100 nm and PDI values below 0.5 for the 30-day study period. The 1:1 ratio produced the largest hydrodynamic size of 175 ± 19 nm (Figure 3a) at the immediate characterisation (0 days). Consequently, the highest PDI of 0.59 ± 0.09 at a volume ratio of 1:1 was also recorded at the 0-day reading (Figure 3b). The NP size and PDI of the 1:1 formulation appeared to reduce and stabilise with time; however, the 1:3 and 1:5 ratios, with larger proportions of the reducing agent present, provided more consistent and stable particle size and PDI results for the duration of the stability study. Statistically significant PDI data were identified for the 1:3 formulation between the day 0 and day 1 measurements (*p* = 0.0412), after which the PDI data appeared more consistent over time. Despite this, the 1:3 and 1:5 groups consistently proved to be monodispersed over the 30 day period.

For the ZP measurement, the only statistically significant results were produced by the 1:1 ratio at the 0-day characterisation point, which also produced the highest ZP value of −6.22 ± 2.45 mV. This measurement exhibited a difference when compared to the 1- (*p* = 0.0009), 7- (*p* = 0.0249) and 14-day (*p* = 0.0050) ZP results (Figure 3c). These statistically significant results can be similarly attributed to the fact that this ratio achieved the largest hydrodynamic size and highest PDI at the 0-day characterisation point and subsequently produced the most unstable result for this sample as well. Subsequent to the 1-day measurement, however, the 1:1 formulation appeared to gradually destabilise over time, particularly between the 1-day and 30-day ZP characterisation points, which produced a statistically significant result (*p* = 0.0182). No statistically significant data were observed amongst the 1:3 and 1:5 groups. The ZP values for these ratios fluctuated along the -30mV range, and the results indicate a slight but insignificant decrease and stabilisation in the ZP value over time, followed by an insignificant increase and destabilisation at the 30-day mark. Previous stability studies performed on SeNPs with the addition of a stabiliser reported comparable results in that the formulation was able to remain stable for more than 30 days; however, it was only able to remain stable for a certain period in the aqueous medium after which the NPs became increasingly unstable [34,35].

### 3.4. Stability of SeNPs in TSB + 2.5% Sucrose

**Figure 4:**
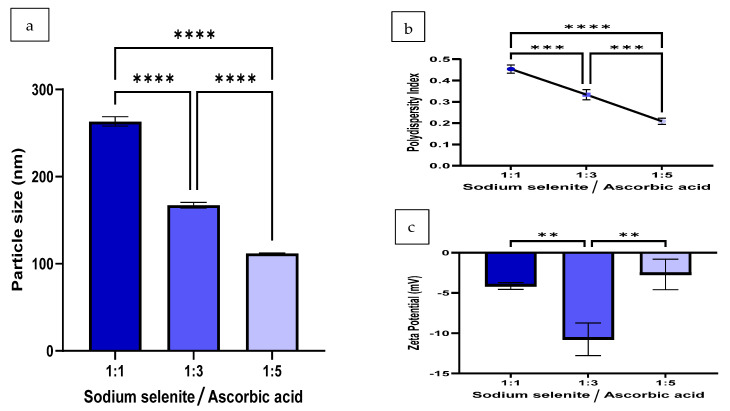
Stability of 1000 μg/mL concentrations of SeNPs in TSB + 2.5% suc. after 24 h of incubation at 37 °C and the effect on (**a**) particle size, (**b**) PDI and (**c**) ZP of SeNPs at different volume ratios of sodium selenite to ascorbic acid (*n* = 3). The synthesis reaction proceeded at 300 rpm, for 30 min, at 25 °C and incorporated 10 mM of SDS as a stabiliser. Statistical significance is represented as follows—**: *p*-value < 0.01, ***: *p*-value < 0.001 and ****: *p*-value < 0.0001. Error bars represent SD.

The DLS analysis revealed that the hydrodynamic size of SeNPs in TSB + 2.5% suc. increased considerably (Figure 4a) in comparison to the particle sizes generated 24 h (1 day) after the formulation (Figure 3a) in dH_2_O. The differences in hydrodynamic sizes produced between 1:1, 1:3 and 1:5 samples among the TSB + 2.5% suc. and dH_2_O samples were 151 nm, 98 nm and 64 nm, respectively. Despite the increase in size displayed by the samples incubated in TSB + 2.5% suc, it appeared that the higher proportion of ascorbic acid rendered the SeNP sample less prone to large particle size increases. This is evident by the linear relationship exhibited in the size differences in relation to the proportion of ascorbic acid present in the formulation.

The SeNPs at a concentration of 1000 μg/mL, suspended in TSB and incubated for a period of 24 h, demonstrated aggregation. Despite the particle size substantially increasing, the PDI only slightly increased (Figure 4b). SeNPs suspended in TSB also displayed a substantial decline in stability, as displayed by all volume ratios exhibiting ZP values above −11 mV (Figure 4c).

### 3.5. Biofilm Forming Ability of S. mutans

**Figure 5:**
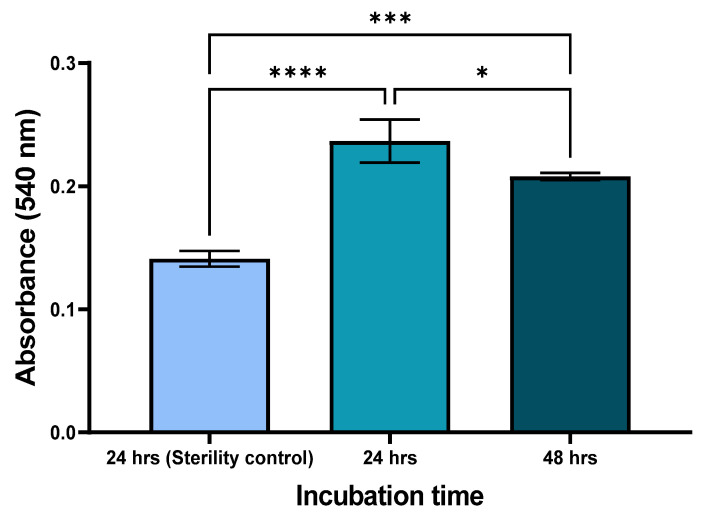
Biofilm formation of *S. mutans* in TSB+2.5% sucrose at 24 and 48 h incubation time periods and sterility control at 24 h incubation as measured by absorbance at 540 nm (*n* = 3). Statistical significance is represented as follows—*: *p*-value ≤ 0.05, ***: *p*-value < 0.001 and ****: *p*-value < 0.0001. Error bars represent SD.

The average absorbance of the biofilm formation of *S. mutans* over a 24 and 48 h incubation period was 0.24 ± 0.014 and 0.21 ± 0.002, respectively (Figure 5). This is slightly less than double the amount of sterility control (24 h), which acquired an average absorbance of 0.14 ± 0.005 (*p* < 0.0001). The biofilm results received over the 48 h incubation period were statistically significant when compared to both the 24 h sterility control (*p* = 0.0007) and the 24 h biofilm formation assay (*p* = 0.0403). According to the biofilm classification described by Christensen et al. (1985) [27], both the 24 and 48 h data results classified the *S. mutans* strain as a weak bordering on moderate biofilm producer.

Li et al. (2013), in a study that tested the sucrose-dependent biofilm formation of three *S. mutans* strains after 24 h incubation against different nicotine concentrations, reported that all three strains exhibited absorbance values below 0.07 at 490 nm [36]. These authors also performed a similar experiment to that reported in this study to test saliva-dependent biofilm formation against nicotine, in which the biofilm growth control exhibited absorbance values below 0.15 at 490 nm for all three *S. mutans* strains [36]. The absorbance results for both experiments appeared to be significantly lower compared to the present study, even though the assays were conducted under similar experimental conditions. Additionally, this indicates that the biofilm assay from this study might be superior as the process of coating wells in saliva was not necessary to produce results within a similar absorbance range.

### 3.6. Effect of Size and Concentration on Antibiofilm Activity of SeNPs

**Figure 6:**
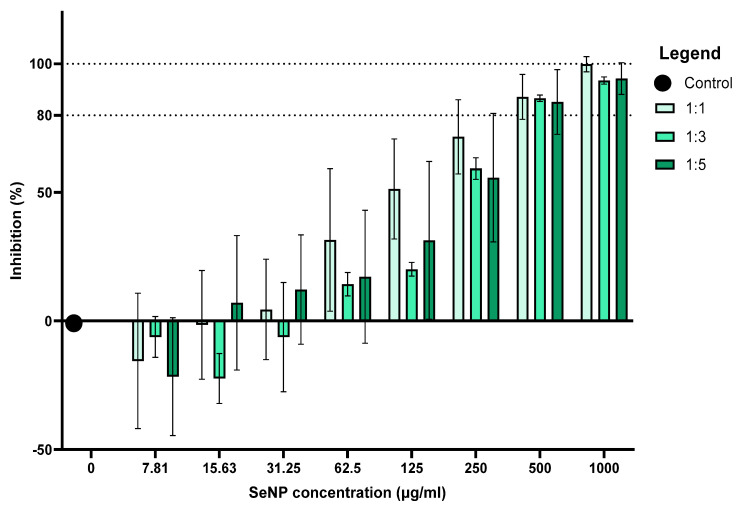
Percentage biofilm inhibition of SeNPs at volume ratios of 1:1, 1:3 and 1:5 and concentrations of 7.81, 15.63, 31.25, 62.5, 125, 250, 500, 1000 µg/mL and 0 µg/mL as the negative control for a 24 h incubation period (*n* = 3). Not all statistically significant comparisons are depicted on the graph. Error bars represent SD.

As shown in Figure 6, the highest biofilm percentage inhibition was achieved at a 1000 µg/mL SeNP concentration, in which the formulation ratio 1:1 of sodium selenite to ascorbic acid generated 99.87 ± 2.41% inhibition relative to the growth control (0 µg/mL) at 0% biofilm inhibition. Volume ratios of 1:3 and 1:5 of sodium selenite to ascorbic acid possessed inhibition percentages comparable to that of 1:1 at the 1000 µg/mL SeNP concentration. Nearly all concentrations that exhibited biofilm inhibition percentages well below 50% (7.81–62.5 µg/mL) demonstrated statistically significant differences, with the SeNP concentrations that exhibited biofilm inhibition percentages appearing well above 50% (250–1000 µg/mL) (*p* < 0.0001). Adeyemo et al. (2022) [29] characterised biofilm percentage inhibition as follows: <0—biofilm growth enhancers; 0–50%—weak antibiofilm activity; and >50%, which were categorised as good biofilm inhibitors [29] and similarly, SeNP concentrations at 125 µg/mL for 1:1 and 250 µg/mL–1000 µg/mL at volume ratios of 1:3 and 1:5 were classified as good biofilm inhibitors. At concentrations below 31.25 µg/mL, 62.5 µg/mL and 15.63 µg/mL were characterised as biofilm growth enhancers for ratios of 1:1, 1:3 and 1:5, respectively [29]. However, another perspective could be that the SeNPs are operating at sub-antibiofilm concentrations and are simply incapable of hindering growth at such low concentrations and, therefore, display concentration-dependant activity.

Similarly, Saeki et al. (2021) reported that sub-inhibitory concentrations of AgNPs (½ MIC, 7.81–31.25 µM) significantly increased (*p* < 0.05) swarming, swimming, twitching motility, and the biofilm formation capacity in *P. aeruginosa* isolates [37]. Among the possible mechanisms theorised in this study include the fact that AgNPs, and more broadly metallic NPs, are responsible for the production of ROS, which might cause oxidative stress [38]. The excess production of ROS ensuing under this oxidative stress may cause adverse effects on cellular components and the destruction of proteins, DNA, and lipids in the microbes [39]. Another potential theory could be that reduced concentrations of metallic NPs can stimulate biofilm development as a defence mechanism against toxicity [40]. This suggests that subinhibitory concentrations stimulate biofilm formation, which accounts for the ‘biofilm enhancer’ classification acquired by the lower concentrations of SeNPs. Additionally, this classification denotes that the *S. mutans* bacterial strain has the potential to operate as a moderate biofilm producer [27].

Several studies have previously reported on the MIC of SeNPs, which have concluded that the MIC against *S. mutans* could be as low as 68 μg/mL [41]. While these studies have reported commendable results at relatively low concentrations, MICs are only a representation of the activity of a drug against bacteria. This study investigated the effect of SeNPs on biofilms, which are clusters of surface-associated bacteria that are embedded in a self-produced matrix. Biofilms are known to be rather formidable, highly resistant, and less sensitive to antimicrobial agents in comparison to planktonic bacteria [42]. It is likely, for this reason, that higher concentrations of SeNPs were required in this study to achieve optimal antibacterial/antibiofilm activity and, as such, ultimately yielded a higher MBIC.

Kwasny & Opperman (2010) proposed that the lowest concentration of an agent that inhibited biofilm growth by ≥80% was considered the MBIC [43]. Hence, an SeNP concentration of 500 µg/mL can be considered the MBIC across all formulations. The biofilm inhibition percentage graph appears to have a directly proportional relationship. This is apparent as the percentage of inhibition tends to increase with the increasing SeNP concentration.

During formulation, the SeNP samples (1000 µg/mL) that were suspended in TSB + 2.5% suc. at molar ratios 1:3 and 1:5 produced better monodispersity and smaller hydrodynamic sizes in comparison to the 1:1 resuspended sample (Figure 4). It is also important to note that no statistically significant comparisons were observed amongst the different volume ratios at concentrations that produced significant antibiofilm activity (500 µg/mL and above). Therefore, it was deduced that the size of these SeNPs during formulation did not greatly affect the antibiofilm activity thereof. Studies have previously reported that antibacterial activity is not significantly impacted by NP size, as much as it may be impacted by the NP concentration or excess production of ROS [44,45]. Similarly, this study displays a directly proportional relationship between the concentration of SeNPs and inhibition of *S. mutans*, but not so much a correlation among the varying particle sizes displayed by each formulation ratio and percentage inhibition.

## 4. Conclusions

This study aimed to investigate the biofilm formation ability of *S. mutans* and determine the antibiofilm activity as well as the MBIC of SeNPs and whether SeNP size influences the biofilm inhibition percentage. SeNPs were as small as 46 ± 4 nm and were found to be monodisperse and relatively stable. SeNPs showed excellent antibiofilm activity against *S. mutans,* up to 99.87 ± 2.41%, and this activity was concentration-dependent. SeNPs are promising candidates for further development as novel therapies for the treatment of periodontitis. Further research could explore the cytotoxicity of their formulation in a mammalian cell line to rule out any potential noxious effects and determine their ability to eradicate preformed *S.mutans* biofilms while also refining the antimicrobial mechanisms of action associated with metallic NPs. Furthermore, future research could experiment on incorporating various drug delivery systems using SeNPs for controlled and localised drug release, such as a hydrogel scaffold, on account of its biocompatibility and comparable physical, chemical and biological properties to that of human tissues. Additionally, this SeNP formulation also exhibits great potential for loading into a niosome, as this system has been known to significantly enhance the biological properties of drugs, such as their antimicrobial and antibiofilm activity.

## Figures and Tables

**Table 1:** Type of biofilm adherence and respective OD criteria required to occupy a specific adherent type [27].

OD Criteria	Biofilm Classification
OD ≤ 2 × OD (control)	Weakly adherent
2 × OD (control) ≤ OD ≤ 4 × OD (control)	Moderately adherent
4 × OD (control) ≤ OD	Strongly adherent

## Data Availability

The data presented in this study are available in this article.

## References

[1] World Health Organisation Oral Health. https://www.who.int/news-room/fact-sheets/detail/oral-health.

[2] Vos T., Abajobir A.A., Abbafati C., Abbas K.M., Abate K.H., Abd-Allah F., Abdulle A.M., Abebo T.A., Abera S.F., Aboyans V. (2017). Global, Regional, and National Incidence, Prevalence, and Years Lived with Disability for 328 Diseases and Injuries for 195 Countries, 1990–2016: A Systematic Analysis for the Global Burden of Disease Study 2016. Lancet.

[3] Tonetti M.S., Jepsen S., Jin L., Otomo-Corgel J. (2017). Impact of the Global Burden of Periodontal Diseases on Health, Nutrition and Wellbeing of Mankind: A Call for Global Action. J. Clin. Periodontol..

[4] Botelho J., Machado V., Leira Y., Proença L., Chambrone L., Mendes J.J. (2022). Economic Burden of Periodontitis in the United States and Europe: An Updated Estimation. J. Periodontol..

[5] Frédéric L.J., Michel B., Selena T. (2018). Oral Microbes, Biofilms and their Role in Periodontal and Peri-Implant Diseases. Materials.

[6] Song W.S., Lee J.K., Park S.H., Um H.S., Lee S.Y., Chang B.S. (2017). Comparison of Periodontitis-Associated Oral Biofilm Formation Under Dynamic and Static Conditions. J. Periodontal Implant Sci..

[7] Vieira Colombo A.P., Magalhães C.B., Hartenbach F.A.R.R., Martins do Souto R., Maciel da Silva-Boghossian C. (2015). Periodontal-Disease-Associated Biofilm: A Reservoir for Pathogens of Medical Importance. Microb. Pathog..

[8] Loesche W.J. (1986). Role of Streptococcus mutans in Human Dental Decay. Microbiol. Rev..

[9] Bowen W.H. (2002). Do We Need to be Concerned about Dental Caries in the Coming Millennium?. Crit. Rev. Oral Biol. Med..

[10] Lemos J.A., Palmer S.R., Zeng L., Wen Z.T., Kajfasz J.K., Freires I.A., Abranches J., Brady L.J. (2019). The Biology of *Streptococcus mutans*. Microbiol. Spectr..

[11] American Academy of Periodontology (2001). Guidelines for Periodontal Therapy. J. Periodontol..

[12] Lertpimonchai A., Rattanasiri S., Arj-Ong Vallibhakara S., Attia J., Thakkinstian A. (2017). The Association Between Oral Hygiene and Periodontitis: A Systematic Review and Meta-Analysis. Int. Dent. J..

[13] Rams T.E., Degener J.E., van Winkelhoff A.J. (2014). Antibiotic Resistance in Human Chronic Periodontitis Microbiota. J. Periodontol..

[14] Joshi D., Garg T., Goyal A.K., Rath G. (2016). Advanced Drug Delivery Approaches Against Periodontitis. Drug Deliv..

[15] Aeran H., Kumar V., Uniyal S., Tanwer P. (2015). Nanodentistry: Is just a Fiction or Future. J. Oral Biol. Craniofac. Res..

[16] Ramburrun P., Pringle N.A., Dube A., Adam R.Z., D’Souza S., Aucamp M. (2021). Recent Advances in the Development of Antimicrobial and Antifouling Biocompatible Materials for Dental Applications. Materials.

[17] Song W., Ge S. (2019). Application of Antimicrobial Nanoparticles in Dentistry. Molecules.

[18] Khan S.T., Ahamed M., Musarrat J., Al-Khedhairy A.A. (2014). Anti-Biofilm and Antibacterial Activities of Zinc Oxide Nanoparticles Against the Oral Opportunistic Pathogens *Rothia Dentocariosa* and *Rothia Mucilaginosa*. Eur. J. Oral Sci..

[19] Hemeg H.A. (2017). Nanomaterials for Alternative Antibacterial Therapy. Int. J. Nanomed..

[20] Filipović N., Ušjak D., Milenković M.T., Zheng K., Liverani L., Boccaccini A.R., Stevanović M.M. (2021). Comparative Study of the Antimicrobial Activity of Selenium Nanoparticles with Different Surface Chemistry and Structure. Front. Bioeng. Biotechnol..

[21] Hosnedlova B., Kepinska M., Skalickova S., Fernandez C., Ruttkay-Nedecky B., Peng Q., Baron M., Melcova M., Opatrilova R., Zidkova J. (2018). Nano-Selenium and its Nanomedicine Applications: A Critical Review. Int. J. Nanomed..

[22] Wadhwani S.A., Shedbalkar U.U., Singh R., Chopade B.A. (2016). Biogenic selenium nanoparticles: Current status and future prospects. Appl. Microbiol. Biotechnol..

[23] Alpaslan E., Geilich B.M., Yazici H., Webster T.J. (2017). PH-Controlled Cerium Oxide Nanoparticle Inhibition of Both Gram-Positive and Gram-Negative Bacteria Growth. Sci. Rep..

[24] Vahdati M., Tohidi Moghadam T. (2020). Synthesis and Characterization of Selenium Nanoparticles-Lysozyme Nanohybrid System with Synergistic Antibacterial Properties. Sci. Rep..

[25] Lin Z.H., Lin F., Wang C.R. (2004). Observation in the Growth of Selenium Nanoparticles. J. Chin. Chem. Soc..

[26] Malhotra S., Jha N., Desai K. (2014). A Superficial Synthesis of Selenium Nanospheres Using Wet Chemical Approach. Int. J. Nanotechnol. Appl..

[27] Christensen G.D., Simpson W.A., Younger J.J., Baddour L.M., Barrett F.F., Melton D.M., Beachey E.H. (1985). Adherence of Coagulase-Negative *Staphylococci* to Plastic Tissue Culture Plates: A Quantitative Model for the Adherence of *Staphylococci* to Medical Devices. J. Clin. Microbiol..

[28] Shinde S., Lee L.H., Chu T. (2021). Inhibition of Biofilm Formation by the Synergistic Action of EGCG-S and Antibiotics. Antibiotics.

[29] Adeyemo R.O., Famuyide I.M., Dzoyem J.P., Lyndy Joy M. (2022). Anti-Biofilm, Antibacterial, and Anti-Quorum Sensing Activities of Selected South African Plants Traditionally Used to Treat Diarrhoea. Evid. Based Complement. Altern. Med..

[30] Li Q., Chen T., Yang F., Liu J., Zheng W. (2010). Facile and Controllable One-Step Fabrication of Selenium Nanoparticles Assisted by L-Cysteine. Mater. Lett..

[31] Chung S., Zhou R., Webster T.J. (2020). Green Synthesized BSA-Coated Selenium Nanoparticles Inhibit Bacterial Growth While Promoting Mammalian Cell Growth. Int. J. Nanomed..

[32] Hei H., Wang R., Liu X., He L., Zhang G. (2012). Controlled Synthesis and Characterization of Nobel Metal Nanoparticles. Soft Nanosci. Lett..

[33] Menamo D.S., Ayele D.W., Ali M.T. (2017). Green Synthesis, Characterization and Antibacterial Activity of Copper Nanoparticles Using L-Ascorbic Acid as a Reducing Agent. Ethiop. J. Sci. Technol..

[34] Yu B., Zhang Y., Zheng W., Fan C., Chen T. (2012). Positive Surface Charge Enhances Selective Cellular Uptake and Anticancer Efficacy of Selenium Nanoparticles. Inorg. Chem..

[35] Bai K., Hong B., He J., Hong Z., Tan R. (2017). Preparation and Antioxidant Properties of Selenium Nanoparticles-Loaded Chitosan Microspheres. Int. J. Nanomed..

[36] Li M., Huang R., Zhou X.D., Gregory R. (2013). Role of Sortase in *Streptococcus mutans* Under the Effect of Nicotine. Int. J. Oral Sci..

[37] Saeki E., Yamada A., Araujo L., Anversa L., Garcia D., Souza R., Martins H., Katsuko R., Kobayashi T., Nakazato G. (2021). Subinhibitory Concentrations of Biogenic Silver Nanoparticles Affect Motility and Biofilm Formation in *Pseudomonas aeruginosa*. Front. Cell. Infect. Microbiol..

[38] Wang L., Hu C., Shao L. (2017). The Antimicrobial Activity of Nanoparticles: Present Situation and Prospects for the Future. Int. J. Nanomed..

[39] Su L.J., Zhang J.H., Gómez H., Murugan R., Hong X., Xu D., Jiang F., Peng Z.Y. (2019). Reactive Oxygen Species-Induced Lipid Peroxidation in Apoptosis, Autophagy, and Ferroptosis. Oxid. Med. Cell. Longev..

[40] Yang Y., Alvarez P.J.J. (2015). Sublethal Concentrations of Silver Nanoparticles Stimulate Biofilm Development. Environ. Sci. Technol. Lett..

[41] Darroudi M., Rangrazi A., Ghazvini K., Bagheri H., Boruziniat A. (2021). Antimicrobial Activity of Colloidal Selenium Nanoparticles in Chitosan Solution Against *Streptococcus mutans*, *Lactobacillus acidophilus*, and *Candida albicans*. Pesqui. Bras. Odontopediatria Clin. Integr..

[42] Shahmoradi S., Shariati A., Amini S.M., Zargar N., Yadegari Z., Darban-Sarokhalil D. (2022). The Application of Selenium Nanoparticles for Enhancing the Efficacy of Photodynamic Inactivation of Planktonic Communities and the Biofilm of *Streptococcus mutans*. BMC Res. Notes.

[43] Kwasny S.M., Opperman T.J. (2010). Static Biofilm Cultures of Gram-Positive Pathogens Grown in a Microtiter Format Used for Anti-Biofilm Drug Discovery. Curr. Protoc. Pharmacol..

[44] Zonaro E., Lampis S., Turner R.J., Qazi S.J.S., Vallini G. (2015). Biogenic Selenium and Tellurium Nanoparticles Synthesized by Environmental Microbial Isolates Efficaciously Inhibit Bacterial Planktonic Cultures and Biofilms. Front. Microbiol..

[45] Zhang L., Jiang Y., Ding Y., Povey M., York D. (2007). Investigation into the Antibacterial Behaviour of Suspensions of ZnO Nanoparticles (ZnO Nanofluids). J. Nanoparticle Res..

